# A Group Intervention for Individuals With Obesity and Comorbid Binge Eating Disorder: Results From a Feasibility Study

**DOI:** 10.3389/fendo.2021.738856

**Published:** 2021-11-03

**Authors:** Trine T. Eik-Nes, KariAnne Vrabel, Jayanthi Raman, Melinda Rose Clark, Kjersti Hognes Berg

**Affiliations:** ^1^ Department of Neuromedicine and Movement Science, Norwegian University of Science and Technology, Trondheim, Norway; ^2^ Stjørdal Community Mental Health Centre, Levanger Hospital, Levanger, Norway; ^3^ Modum Bad Psychiatric Center, Vikersund, Norway; ^4^ Graduate School of Health, University of Technology Sydney, Sydney, NSW, Australia

**Keywords:** eating disorder, binge eating disorder (BED), intervention, feasibility, design, psychological comorbidity

## Abstract

**Purpose:**

A common challenge among a subgroup of individuals with obesity is binge eating, that exists on a continuum from mild binge eating episodes to severe binge eating disorder (BED). BED is common among bariatric patients and the prevalence of disordered eating and ED in bariatric surgery populations is well known. Conventional treatments and assessment of obesity seldom address the underlying psychological mechanisms of binge eating and subsequent obesity. This study, titled PnP (People need People) is a psychoeducational group pilot intervention for individuals with BED and obesity including patients with previous bariatric surgery. Design, feasibility, and a broad description of the study population is reported.

**Material and Methods:**

A total of 42 patients were from an obesity clinic referred to assessment and treatment with PnP in a psychoeducational group setting (3-hour weekly meetings for 10 weeks). Of these, 6 (14.3%) patients had a previous history of bariatric surgery. Feasibility was assessed by tracking attendance, potentially adverse effects and outcome measures including body mass index (BMI), eating disorder pathology, overvaluation of shape and weight, impairment, self-reported childhood difficulties, alexithymia, internalized shame as well as health related quality of life (HRQoL).

**Results:**

All 42 patients completed the intervention, with no adverse effects and a high attendance rate with a median attendance of 10 sessions, 95% CI (8.9,9.6) and 0% attrition. Extent of psychosocial impairment due to eating disorder pathology, body dissatisfaction and severity of ED symptoms were high among the patients at baseline. Additionally, self-reported childhood difficulties, alexithymia, and internalized shame were high among the patients and indicate a need to address underlying psychological mechanisms in individuals with BED and comorbid obesity. Improvement of HRQoL and reduction of binge eating between baseline and the end of the intervention was observed with a medium effect

**Conclusion:**

This feasibility study supports PnP as a potential group psychoeducational intervention for patients living with BED and comorbid obesity. Assessments of BED and delivery of this intervention may optimize selection of candidates and bariatric outcomes. These preliminary results warrant further investigation *via* a randomized control trial (RCT) to examine the efficacy and effectiveness of PnP.

## Introduction

One common condition among a subgroup of individuals within the obesity^1^ population is a type of disordered eating behavior called binge eating. Binge eating behavior consists of eating objectively large volumes of food within a discrete period of time and exists on a continuum from mild binge eating to the more severe, binge eating disorder (BED) (1). BED is a psychiatric disorder that is characterized by recurrent episodes of binge eating, in the absence of compensatory behaviors and accompanied by a sense of loss of control (2). BED typically manifests in elevated body weight, greater preoccupations with food, poor dietary restraint, body dissatisfaction, psychological distress, low self-esteem, guilt, and self-disgust over food consumption (3–6). Whilst the condition has only recently been recognized by the Diagnostic and Statistical Manual fifth edition (7) as its own defined category, BED represents the most common eating disorder (ED) with prevalence rates ranging from 0.2% to 6.6% of the worldwide population (8–10). Co-morbid emotional disorders and cognitive deficits have been proposed to underlie and maintain numerous BED symptoms and difficulties including regulating food intake, generating alternative behavioral patterns as well as following through with meal plans and treatment recommendations (11–14). Due to the nature of binge eating, such behavior contributes to the progressive escalation of Body Mass Index (BMI) over time. Between 30-50% of individuals with obesity who seek weight management treatment report clinically significant degrees of binge eating (15–17), for example, binge eating episodes that last a full day (18–20). Moreover, a large US study found that 42% of those with a lifetime BED had obesity when the study was performed (21). It has furthermore been shown that adults with BED and bulimia nervosa are more likely to have been categorized with severe obesity compared to individuals without ED (22). More, in a recent health survey from England, individuals with obesity were more likely to screen positive for a possible ED, compared with those in lower BMI categories (23).

BED is common among bariatric patients (24) and the prevalence of disordered eating and ED in bariatric surgery populations is well known (25–28). Further, weight regain in patients with ED has been shown in post-surgical bariatric patients (29, 30) with one study showing pre surgery disordered eating in 65% of patients with weight regain (29). As patients with bariatric surgery can be helped with restriction of meal and portions post-surgery, some phenotypes may develop various eating disordered behaviors, such as grazing, as a compensatory mechanism, resulting in a higher energy intake (31). The association between the disordered behavior grazing (i.e., eating small portions of food with and without loss of control) and post-surgical weight regain (32), suggest a need for assessment and treatment of disordered eating such as binge eating and grazing pre- and post-surgery. Thus, comprehensive pre- and post-surgery assessments and interventions for disordered eating such as binge eating, and grazing may optimize outcomes of both conservative obesity treatments and bariatric outcomes.

There is ample evidence to show that BED is associated with elevated rates of psychiatric co-morbidity and lower quality of life (8, 33). When compared with individuals with obesity but without binge-eating problems, individuals with BED report earlier onset of obesity, more frequent episodes of dieting, marked weight fluctuations, more severe obesity (34), elevated body weight, greater preoccupations with food, poor dietary restraint, body dissatisfaction, psychological distress, low self-esteem, as well as guilt and self-disgust over food consumption (3–6). They also report less self-esteem, more depressive symptoms, less eating restraint, greater overall psychological distress and a greater lifetime prevalence of psychiatric disorders than do obese individuals who do not binge eat (35). Several empirical studies indicate that obese individuals who binge eat have higher rates of Axis I and Axis II mental disorders than obese individuals who do not binge eat (21, 36).

Despite the notion that difficulties with poor body image and associated stressors such as weight stigma have been shown to be commonly experienced by individuals with BED (37) and obesity (38, 39), it is rarely addressed in treatment approaches for the condition. Wang, Jones (40) reinforced need for increased targeting of overvaluation of shape and weight as it was shown to be a core symptom in patients seeking treatment for BED. Although current treatment approaches for obesity and BED target overvaluation of weight and shape, few interventions acknowledge the intricate relationship between binge eating, weight stigma and overvaluation of weight and shape. There are few studies which have examined BED stigma (41), and the majority of obesity interventions are concentrated on weight loss, lacking consideration of weight stigma (42). Hence, the damaging consequences of weight stigma (43) are seldom addressed in the treatments of obesity or BED.

Existing treatments recognize the mechanisms between ED and attachment functioning (44), and the psychological constructs that may contribute to development and maintenance of binge eating behavior including environmental, social, biological, physiological, political, and familial stressors (45). The prevalence of attachment insecurity among individuals with ED is shown to be high (46–48). Individuals with BED have been reported to have an avoidant attachment style, thus, individuals who are insecurely attached, may be more overwhelmed when they experience distress (49) compared to those securely attached.

Individuals with BED have shown to benefit from cognitive-behavioral therapy (CBT) and pharmacological treatments such as second-generation antidepressants, lisdexamfetamine, and topiramate (50, 51). As individuals with BED often present for weight loss treatments rather than treatment for an ED (4, 52), the condition is associated with a persistent pattern of symptom remission and relapse (53). Currently, CBT has been established as a beneficial psychological modality to treat the condition (54, 55).

Even though BED symptoms have been demonstrated to reduce following CBT, research has shown that this treatment modality is only partially effective (56, 57). Consequently, many individuals remain symptomatic or experience symptom reoccurrence following the conclusion of CBT treatment (58, 59). Other non-invasive approaches include behavioral weight loss interventions, guided-self-help, and interpersonal psychotherapy (IPT). However, similarly with CBT treatment, each have been established to be only partially effective, produce unsustainable effects, and/or not address core ED symptoms (5, 54, 60).

The recommended treatment modality for patients suffering from both obesity and BED is CBT (52), but IPT has also been shown to be acceptable to patients with BED (61, 62). Small effects have been observed in improved behavioral symptoms at posttreatment in favor of CBT in comparison to IPT, but extant studies have lacked comparison of the treatment modalities at follow-up (63). Other integrated treatment for patients with obesity and BED has been developed and tested (64–66).

However, high rates of attrition from interventions are reported both in patients with ED (67–69) and patients with obesity seeking weight loss treatments (70, 71).

The complex nature of obesity (72–74) and co-morbid ED (75) suggest prevention and treatment may be improved by addressing multiple factors contributing to obesity rather than just targeting weight (76). Recent studies recommend assessment of psychological distress, interpersonal sensitivity, shape-weight concern, and internalized weight stigma in patients seeking treatment for obesity (77). As many patients seeking treatment for obesity also suffer from BED, it is likely that a large group of patients with BED and obesity are not detected in obesity treatment settings.

Together these results highlight clear clinical implications and a need for innovative directions in the treatment of obesity and comorbid BED.

This study aimed to describe the rationale, design and feasibility of an intervention for patients with obesity and comorbid BED in an outpatient setting in Norway. This pilot study was conducted to inform the design of a larger clinical trial and to provide direction as to whether a larger study on treatment of obesity and comorbid BED should be conducted (78). The main purpose of this pilot study was to investigate feasibility by tracking attendance, adverse events related to the intervention and patient attrition. Secondly, we aimed to assess symptom severity of ED behaviors and quality of childhood, alexithymia, overvaluation of weight and shape, body image perception and internalized shame in patients with obesity and comorbid BED at baseline. Third, we aimed to evaluate change in frequency of binge eating episodes and health related quality of life (HRQoL) from pre to post intervention.

## Material and Methods

### Study Design

This pilot study was an open label pilot in a naturalistic setting to evaluate feasibility of a study population that is more representative of the population at large compared to randomized trials. Patients stayed on prescribed medication (treatment as usual) during treatment. All patients were recruited from a specialized obesity outpatient unit in a tertiary care hospital that included adult patients (≥18 years) to a mental health outpatient clinic during a period from November 2018 to August 2020. All participants except 5 patients were classified with morbid obesity (BMI ≥40 kg m2 or BMI≥35 kg m2 with comorbidity) where 4 of these patients had a history of bariatric surgery. After assessment of BED, 42 patients were in five consecutive treatment groups included in the pilot study where they received a 10-week group intervention. Recruitment ended when the fifth treatment group was completed in November 2020.

### Participants

Patients who in the medical consultation at the obesity clinic were identified as having symptoms of BED (objective large binge episodes and loss of control) and considered likely to benefit from treatment of BED were referred to a dedicated BED team at the mental health outpatient clinic.

Exclusion criteria were those not able to read or speak Norwegian, ongoing psychosis, current substance abuse, current alcohol abuse, severe suicidality, and those with severe impairment due to mental or physical health which could interfere with the ability to comply with attending treatment (3-hour long therapy session for a duration of 10 weeks).

The study was approved by the Regional Ethical Review Board of Mid-Norway and the data access committee at Nord-Trøndelag Hospital Trust. Patients received oral and written information about the study at inclusion from their therapist during their first assessment session. All included patients signed an informed consent form. Patients were informed that they could withdraw their consent at any given moment without any consequences for their ongoing treatment.

### Treatment

The framework of the “People Need People” (PnP) intervention is the patient’s attachment functioning with a dual focus on BED symptoms. The content of PnP is based on Bowlby’s attachment framework (79) as higher levels of attachment insecurity are related to greater ED symptoms (80, 81). The intervention targets binge eating behavior in the framework of attachment functioning and ED (44), as the prevalence of attachment insecurity among individuals with eating disorders is shown to be high (46–48). Further, the intervention considers possible underlying emotional difficulties in individuals with BED such as alexithymia (inability to describe and/or recognize one’s own emotions) (82), shame (83) and/or attachment insecurity (80, 84) in the development and maintenance of disordered eating pathology, making such difficulties relevant treatment targets.

In this intervention, binge eating episodes and subsequent obesity is understood as a response to stress (85) caused by internal (e.g., overvaluation of weight or shape) or external stressors (e.g., weight stigma). In the intervention, the responses (binge eating or grazing) to stressors are linked to the physiological reactions “fight, flight or freeze” that can occur in response to stressors (86, 87). Moreover, patients are presented with empirical evidence and experiences explaining why binge eating behavior in response to stress often is associated with increased food intake or a shift toward a higher fat and sugar diet (88). The intervention places emphasis on the body’s stress response system (the hypothalamic-pituitary-adrenal [HPA] axis) (89–91), which may explain binge eating behavior and subsequent weight gain (92). Patients learn that eating both sugar and fat can reduce the extent of stress-induced activation of the HPA axis (90, 92) and thus explain why and how binge eating occurs. The conceptualization of binge eating in response to stress in humans can also be seen in animals who eat both lard and sucrose when stressed (92).

The main goal of the intervention is understanding and learning other responses to stressors than binge eating behavior when faced with internal or external stressors, aimed at fostering the patient’s use of *other people* as a “safe haven”.

The PnP intervention is designed by the first and the last author of the study, and is a 10-week psychoeducational group intervention, administered by two therapists over 10 weeks in 3 hours sessions. All sessions are led by two therapists and include three parts including (1) a didactic section using a PowerPoint presentation using audio and visual forms of teaching (2) with small-group learning activities (in pairs or small groups) and whole-group discussion with reflections and (3) eating lunch together. Interventions were weekly as studies indicate that regular therapy attendance for patients receiving a shorter course of therapy such as the PnP intervention is associated with better final outcomes (93). Further, a large study investigating session frequency found more clinically meaningful improvements in clients receiving weekly sessions compared to those attending every two weeks (94). Moreover, we wanted to avoid infrequent scheduling to avoid patients being less actively involved with the therapy and feeling less connected with group members and therapists.

The PnP intervention targets several underlying psychological constructs that patients may consider contributory to the development and maintenance of binge behaviors (45, 95) affecting unhealthy weight gain. Influencing stressors (for example poor body image, adverse childhood events, weight bias, internalized weight bias, competing demands of personal self-care versus care of family members, and challenges of interpersonal relationships) are presented in the modules with small group learning activities.

The intervention does not include dietary modules, nor physical activity modules. The content for the intervention is based on a psychoeducational framework with consideration of ED mental health literacy (96). The intervention include several constructs of BED as interventions program success will increase with the number of elements targeted (97).

The intervention is not body-weight-centric and aims to treat BED to decrease ED pathology and facilitate a healthier lifestyle. Program content, details of each module and goals are outlined in **Table 1**.

**Table 1:** PnP content.

Module	Theme	Goals
**1**	Introduction	Acknowledgement of privilege from therapists Expectations Goals of this treatment Create a content of safety
**2**	Eating disorders Binge eating disorder	What is an eating disorder? Binge eating disorder (BED) BED and weight The function of food
**3**	Attachment (part 1)	Attachment and our basic needs Emotions and food Importance of others and how this relates to food and body image Attachment and food The Hi-game*
**4**	Attachment (part 2)	The body’s stress response system HPA**/fight, flight, freeze Adverse childhood experiences External stressors/chronic stress and food/weight Regulating emotions with food
**5**	Stigma and shame (part 1)	What is shame? Internalized shame «Compass of shame» Desire for weight loss
**6**	Stigma and shame (part 2)	Weight stigma Stigma in health care Internalized weight stigma Experiences of shame
**7**	Overvaluation of shape and weight	Body Image/body hatred Body’s functioning Feeling (un)safe in my body
**8**	Self assertion/Boundaries	The importance of “boundaries” Care giver needs/(not) being cared for The importance and challenges with “others”
**9**	Loved ones *2 hr information session with chosen loved ones*	Understanding of binge eating disorders, dieting and shame. Diet talk The experience and role of care givers
**10**	Summary	What was your goal? What will be important moving forward? What has been meaningful?

*The talking tool “the Hi-game” is provided to the patient for use with families/loved ones to support and facilitate conversations related to feelings with emphasis on the importance of others.

** hypothalamic-pituitary-adrenocortical.

The dedicated BED team included a psychiatrist, psychologist, and certified health personnel with a bachelor’s degree (physiotherapist, occupational therapist, and mental health nurses with > 10 years’ experience). All had experience and training in assessment and treatment of mental health illnesses, in particular ED and childhood trauma with broad experience in psychoeducation, psychotherapy and/or group therapy. Moreover, all therapists received bi-weekly supervision by a trained psychiatrist and an experienced psychologist. The assessment and treatment were performed according to standard clinical psychiatric care in Norway.

## Methods

### Screening

Weight and height were measured as part of the routine clinical practice at the obesity clinic. After referral from the obesity outpatient clinic, a first clinical assessment was performed by one of the members from the BED team. In addition, a second clinical assessment of body image and overvaluation of shape and weight was conducted by a physical therapist. The construct “body image disorder” is complex and multidimensional (98) and includes several aspects such as overvaluation of shape and weight, body dissatisfaction, shame, impaired interoceptive awareness and lack of familiarity with one’s own body (99). In the assessment, we sought to cover these aspects through an interview, questionnaires and two clinical tests. Overvaluation of shape and weight and perception of body size is presented in this article. Theoretical foundation and data on the participants’ body image disorder with more in-depth data on emotional bodily experiences will be presented in a separate paper.

The information gathered in the two clinical assessments were discussed and evaluated by the BED team. Diagnosis of BED was done according to diagnostic criteria using Diagnostic and Statistical Manual of Mental Disorders (DSM-5) (2).

All patients, except those with previous bariatric surgery (N=6), were diagnosed with BED according to DSM-5 criteria. Those with previous bariatric surgery were included in the pilot study even if they did not meet all the DSM-5 BED criteria and were hence diagnosed with Other Specified Feeding and Eating Disorder (OSFED); DSM-5). Patients with a history of bariatric surgery were included as their ability to eat larger amount of foods was limited because of their bariatric surgery. All these 6 patients identified with the 1b) criterion “sense of lack of control overeating during the episode (e.g., a feeling that one cannot stop eating or control what or how much one is eating)”. Further, all 6 patients would according to the clinical interview and medical records meet the 1a) criteria: “Eating, in a discrete period of time (e.g., within any 2-hour period), an amount of food that is definitely larger than most people would eat in a similar period of time under similar circumstances” prior to their bariatric surgery. Additionally, all 6 patients who previously had bariatric surgery described patterns of disordered eating with loss of control, grazing or use of alcohol as a compensatory mechanism for lack of being able to binge eat post-surgery. There were no data describing a diagnosis of BED prior to bariatric surgery in their medical records.

### Measures

Baseline data included weight and height measurement, and a package of self-report questionnaires for BED pathology, body image and underlying psychological constructs.

#### Weight and Height

Patients’ weight was at baseline measured with the scale InBody 720 (Biospace, Korea). InBody 720 is commonly utilized in Norwegian clinical practice for treatment of obesity. Patients were dressed in light clothing without shoes.

### Feasibility

Attrition was evaluated after 10 weeks where date and number of treatments were reported from their medical records. Drop out was defined if a patient or therapist initiated discharge prior to 10 weeks. Other feasibility measures (100) such as acceptability, demand, implementation, integration and expansion related to the PnP intervention will be presented in a separate paper.

#### The COOP/WONCA Charts

The COOP/WONCA charts (101) - the Norwegian version (102) measure six core aspects of HRQoL (α=0,84). The questionnaire is also used to assess functional status as it has previously been used to assess change in functional status over time, and for measuring outcomes of interventions. The questionnaire asks the patient’s status during the past two weeks in the dimensions physical fitness, feelings, daily activities, social activities, changes in health and health condition. The response categories are scored from 1 to 5, and higher scores indicate worse HRQoL. Each item is rated on a five-point ordinal scale ranging from 1 (“no limitation at all”) to 5 (“severely limited”). The five response choices of the Coop/Wonca charts were at baseline stratified into three categories: score 1–2 corresponding to better HRQoL; score 3 corresponding to neutral/middle HRQoL; score 4–5 corresponding to the low HRQoL. The dimensions have correlated well with other measures of physical and emotional functioning (103).

#### The Eating Disorder Examination-Questionnaire-6.0

The Eating Disorder Examination-Questionnaire-6.0 (EDE-Q 6.0) (104) was administered at baseline using the Norwegian version (105) to report a global score of ED pathology (α=0.89) with different domains of disordered eating (restraint α=0,79), weight concerns (α=0,70), shape concerns (α=0,78), eating concerns (α=0,76) and frequency of key ED behaviors (e.g., objective binge eating). The global attitude score is calculated from the subscales. The EDE-Q has shown to be adequate and suitable for use with patients with BED (106).

#### Clinical Impairment Assessment

Severity of psychosocial impairment due to ED was measured using the Norwegian version (107) of the Clinical Impairment Assessment (CIA) (108). The scale also provides a continuous global score and three sub-scale scores (Personal impairment, Cognitive impairment, and social impairment). A threshold global score ≥16 is associated with “caseness” (107). At baseline we assessed the global score (α=0,90) and severity of impairment using the three sub-scale scores: personal impairment (α=0,88), cognitive impairment (α =0,90) and social impairment (α, = 0,78).

#### Repetitive Eating Questionnaire

Grazing was at baseline assessed with the Repetitive Eating Questionnaire (REP-Q) (109) using the Norwegian version of the Repetitive Eating Questionnaire (110). The measurement is an indicator of presence or absence of compulsive and repetitive eating (eating in a distracted or mindless way). At baseline, we assessed the total score (α= 0,69) and the sub-scales compulsive eating (α =0,77) and repetitive eating (α =0,52).

#### Internalized Shame Scale

Internalized shame was at baseline assessed with the Internalized Shame Scale (ISS) (α= 0,85) (111), using the Norwegian version (112). ISS is a self-report measuring tapped by 24 global self-evaluative items and is focused on evaluating the extent to which the negative affect of shame becomes magnified and internalized. The instrument has shown to be reliable and valid instrument in general populations and in clinical populations with high internal consistency between items (113). A score of 50 is suggested as a cut-off for problematic levels of shame (112).

#### Single Item Childhood Experience Question

To assess whether adverse childhood events would be of importance, a single item childhood experience question was phrased: ‘When you think about your childhood, would you describe it as’: ‘Very good–good–average–difficult–very difficult’ referring to the patient’s subjective, global perception of his/her childhood. Those who reported their childhood as difficult or very difficult were categorized with “poor quality of childhood”. The overall quality of the single item question of childhood has earlier been used in large Norwegian public health study (the HUNT study) (114), and Vederhus, Timko (115) indicated that the question would could be an empirically supported method of assessing adverse childhood events (ACEs).

#### Toronto Alexithymia Scale

The Toronto Alexithymia Scale (TAS-20) was used to assess patients’ ability to identify, regulate and express one’s own emotions (alexithymia) (Bagby, Parker, & Taylor, 1994) using the Norwegian version (116). We assessed a total score of TAS-20 (α= 0,84) and the following subscales: difficulty identifying feelings (α=0,71), difficulty describing feelings (α=0,77) and externally oriented (a way to avoid feelings) (α=0,65). We used the following cut-off score for alexithymia: < 50 = non-alexithymia, > 61 = alexithymia (117).. Internal consistency has been reported to be high with α’s >80 (118, 119).

### Overvaluation of Shape and Weight

Assessment of overvaluation of shape and weight was gathered using the two items from the validated Norwegian version of EDE-Q: *“Over the past 4 weeks, has your shape influenced how you feel about (judge/think/evaluate) yourself as a person?”* and *“Over the past 4 weeks, has your weight influenced how you feel about (judge/think/evaluate) yourself as a person?”* (120). These items were described by the physical therapist to ensure that patients understood the nature of the questions. The items were rated on a seven-point severity scale ranging from 0 to 6 where 0 represents the absence of the weight or shape concern in the question and 6 represents its presence to an extreme degree (104, 121). The 2 questions have earlier been used to define overvaluation of shape and weight in participants with ED (122, 123). Mean scores for the abovementioned items were used to determine a proxy for overvaluation of shape and weight. Patients who reported a score ≥ 4 on any or both items were defined as overvaluating weight and shape (124).

### Body Image Perception

**Figure 1:**
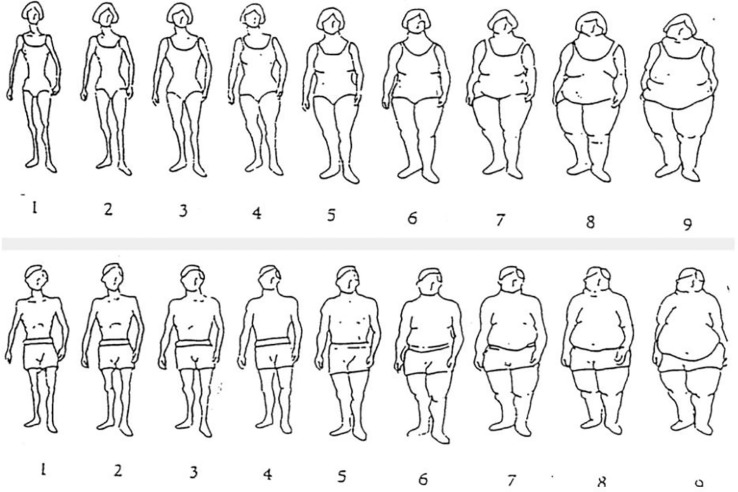
Stunkard’s silhouette body drawings *Permission to use the figure is granted by the original author.

In addition to assessing overvaluation of weight and shape, we assessed three constructs of body image perception at baseline. We measured (1) (mis)perception of one’s own body and (2) desired body and (3) variation of experience of body size using Stunkard’s silhouette body drawings (125) (see **Figure 1**) using nine silhouettes that represent an ordinal scale with increasing figural scales. Patients were asked to indicate which figure best depicted the cognitive representation of their own size (*“Your perceived actual size”*); second, they were asked to indicate which figure best depicted their desired body size (*“your ideal body size, the size you would prefer to have”*). Their actual body size figure was assessed by the physical therapist in the clinical assessment using the silhouette scale. Finally, they were asked: *“does your experience of body size use to vary so much that you want to describe it with several figures*?” and *“what is the variation of figures?*” using the silhouette scale, depicting their variation of their affective evaluation of their size. Differences between actual and perceived was estimated to describe differences between the subjective measures of the participant ‘s own body/figure and actual size. Differences between perceived size and desired body size (body size misperception) was estimated along with variation of figures.

Frequency of binge eating episodes from the frequency measures in EDE-Q, and HRQoL using the Coop-Wonca charts was measured pre- and post-intervention. All other self-report questionnaires were measured at baseline to describe symptom severity in the study population.

## Statistical Analysis

Descriptive frequency analyses were conducted on the quantitative data with respects to adherence, ED pathology, quality of childhood, alexithymia, internalized shame, impairment due to ED, body dissatisfaction and HRQoL. We tested normality of data distribution using the Shapiro-Wilk test which indicated that the HRQoL data were not normally distributed. To test whether there were significant differences between binge eating episodes and HRQoL pre- and post-intervention, we thus used Wilcoxon signed rank test. We calculated the r effect size for Wilcoxon signed-rank tests (126) (small r ≥.10, medium r ≥.30, large r ≥.50) (127). We noted whether correlations reached a medium effect size (i.e., r ≤.3). All analyses were conducted in IBM SPSS (version 27).

## Sample Size Calculation

The pilot study aimed to include 40 patients. We did not perform a power calculation due to the feasibility nature of the study. However, the sample size was based on guidelines suggesting sample sizes in feasability studies to be at least 30 patients to estimate a parameter (128). Further, we based our sample size on other BED treatment pilot studies varying from 7 to 41 participants (129–131). Lastly, Moore, Carter (78) suggested that at least 12 patients is recommended for both practical reasons and for providing preliminary data informing later design estimate average values and variability for a future larger clinical trial,

## Results

### Participant Characteristics

Patients were 36 females (85.7%) and 6 (14.3%) males with a mean age of 38.7 years (SD=12.8) (**Table 1**). Mean BMI was 42.4 (SD=7.1), with lower BMI levels among those with previous bariatric surgery (M=33.4. SD=3.5). Four of the five patients without morbid obesity, had a history of bariatric surgery. More than half (66.6%) of the patients had a partner, spouse, or a boyfriend/girlfriend. Those who at baseline were single (33.4%) included those without a partner, spouse, or boyfriend/girlfriend (separated, divorced, widow/widower).

### Feasibility

A total of 67 adult patients were referred from a tertiary care obesity outpatient clinic to an outpatient mental health centre for assessment of BED during the study period. A total of 25 patients (37.3%) of those referred, 20 females and 5 males, did not receive the intervention. The reasons why these patients were not given the intervention were due to not meeting the DSM-5 criteria for BED (n=3), cancelling the initial appointment for assessment of BED (n =3), not wanting to receive treatment for BED (n =10), severe impairment due to comorbid mental health disorders (e.g. Asperger’s syndrome or personality disorder (n =3), parallel treatment received at other mental health services (n =3) or no indication of mental health illness in the referral or moved to another part of the country (N=3), leaving us with 42 patients receiving the 10-week PnP group intervention.

None of the 42 patients who received the PnP intervention dropped out of the treatment during the 10 weeks of treatment, with a median attendance of 10 sessions, 95% CI (8.9,9.6) and 0% attrition. No adverse events were reported during or after the intervention.

### Symptom Severity

EDE-Q Global Scores at baseline (M =3.9, SD =0.9) were high, suggesting high levels of ED psychopathology (optimal cut-off score of 2.50 and 3.26 when BMI ≥ 30 kg/m^2^) (120). Further, high levels in different domains of ED pathology (dietary restraint, eating concern, weight concern, shape concern) were observed among the participants (**Table 2**).

**Table 2:** Baseline characteristics.

Variables		
	**N**	**Percentage**
**Gender**	42	
** Male**	36	85,7
** Female**	6	14,30
**Marital status**	42	
** Married/partnership**	28	66,6
** Single/divorced/separated/widow**	14	33,4
		**M (SD)**
**Age (years)**		38,7 (12,8)
**BMI (kg/m2)**		42,4 (7,1)
**No bariatric surgery (n=36)**		43,8 (6,5)
**Previous bariatric surgery (n=6)**		33,4 (3,5)
**EATING DISORDER PATHOLOGY**	35	**M (SD)**
** GLOBAL EDE-Q SCORE**		3.9 (0.9)
** Dietary Restraint**		2.3 (1.3)
** Eating Concern**		3.4 (1.3)
** Weight Concern**		4.3 (1.0)
** Shape Concern**		4.8 (1.0)
** IMPAIRMENT**	41	**M (SD)**
** CIA global score**		29.5 (9.0)
** Personal impairment**		15.1 (3.9)
** Social impairment**		7.6 (3.6)
** Cognitive impairment**		6.9 (3.9)
** Grazing**	42	**M (SD)**
** Total REP-Q score**		3.8 (1.2)
** Compulsive subscale**		4.0 (1.1)
** Repetitive eating subscale**		3.6 (1.7)
**OVERVALUATION OF WEIGHT AND SHAPE**	41	
** Has your weight influenced how you think about (judge) yourself as a person?**		5.27 (1.2)
** Has your shape influenced how you think about (judge) yourself as a person?**		5.22 (1.3)
**CASENESS OF OVERVALUATION OF WEIGHT AND SHAPE**	41	**Percentage**
** Overvaluation of weight (>4)**		92.7%
** Overvaluation of shape (>4)**		90.2%
** Overvaluation of both weight and shape (>4)**		90.2%
** Overvaluation of both weight and shape (>4)**		90.2%
**ALEXITHYMIA (TAS-20)**		**M (SD)**
** Total score**	40	60.2 (11.6)
** Identifying emotions**	40	23.4 (5.3)
** Describing feelings**	40	13.2 (3.3)
** Externally oriented thinking**	40	21.2 (5.1)
**Quality of childhood**	40	**Percentage**
** Very difficult/difficult**	23	57.5
** Average**	7	17.5
** Very good/good**	10	25.0

Data given as mean ± SD or N (%).

Participants also reported high levels of grazing (**Table 2**). Total REP-Q mean score was 3.8 (SD= 1.2) with a mean score of 4.0 (SD=1.1) on the compulsive subscale and a mean score of 3.6 (SD=1.7) on the repetitive eating subscale (eating in a distracted or mindless way). Patients with previous bariatric surgery reported higher levels of grazing with a total REP-Q mean score (N=6) of 3.7 (SD=1.2) and a mean score of 4.3 (SD=1.0) on the compulsive subscale and a mean score of 4.2 (SD=1.1) on the repetitive eating subscale (eating in a distracted or mindless way).

High severity of psychosocial impairment due to ED was observed with a mean of 29.5 (SD 9.0) using the CIA. The majority (92.7%) of patients scored above the threshold score (sum score ≥16) associated with “caseness” (a term used to define a clinical case). Results from the three domains of CIA are shown in **Table 2**.

Patients reported high levels of internalized shame with a mean score of 72.3 (SD=14.4). Thus, the majority (90%) of all participants reported problematic levels of shame (score ≥50).

More than half (57.5%) of the patients reported their quality of childhood to be poor (**Table 2**). Additionally, alexithymia, measured with TAS-20, was significant with a mean score was 60.2 (SD=11.6). Hence, 45.0% of the participants scored above the suggested cut off score (≥61) for alexithymia. **Table 2** additionally shows data from the domains of TAS-20.

### Health Related Quality of Life

**Figure 2:**
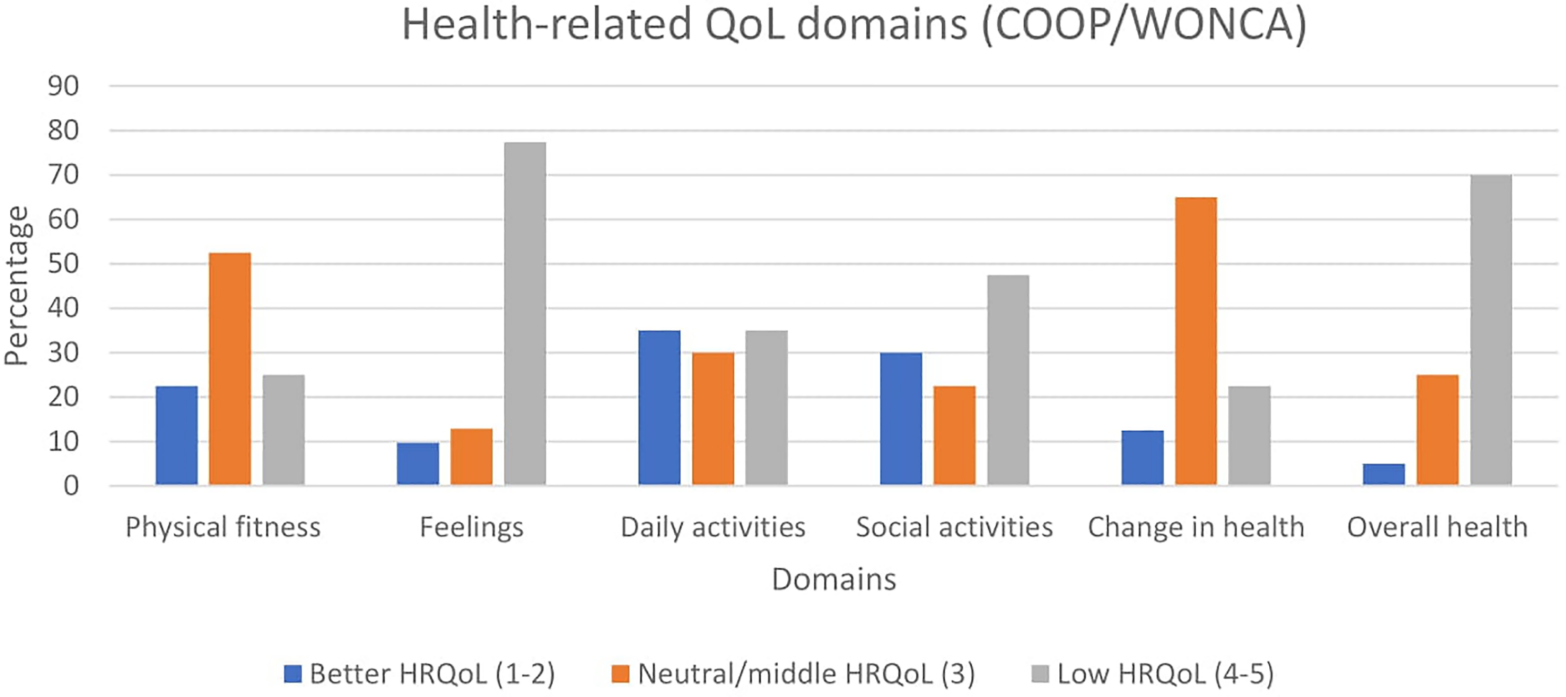
Distribution of HRQOL at baseline (Coop/Wonca). The Five response choices of the Coop/Wonca charts were stratified into three categories: score 1-2 corresponding to better HRQoL; score 4-5 corresponging to the low HRQoL.

The HRQoL among the patients was generally low at baseline (Table IV) and varied according to the dimensions of HRQoL (physical fitness, feelings, daily activities, social activities, and general health). Stratification of the five response choices of the Coop/Wonca charts showed particularly low HRQoL in the dimensions “feelings” and “social activities (**Figure 2**). In total, 77.4% of all patients reported to be “quite a bit or extremely” bothered by emotional problems, and 47.5% of the patients reported that their physical or mental health limited their social activities with family, friends, neighbors or groups. 70% of participants rated their overall health to be low (bad/very bad) at baseline. Distribution of categories of HRQoL at baseline are shown in **Figure 1**.

### Overvaluation of Weight and Shape

Cognitive symptoms of overvaluation of weight and shape was very high indicating that both overvaluation of weight and shape was present to an extreme degree among the participants (**Table 2**).

In the assessment of body image perception using the Stunkard’s silhouette body drawings, 66.7% of the patients perceived their body size accurately. None of the patients reported to perceive their body size below figure # 6. Difference in body size perception from actual size differed with 2 sizes in those who underestimated (20.5%) or overestimated (12.8%) their bodies’ sizes. In general, all patients reported their ideal body figure to be smaller than their perceived figure. In total, 71.1% indicated a large difference (≥ 3 figures) between their ideal body figure and perceived body figure. Of all patients, 54.1% experienced that their body size varied so much that they wanted to describe it with several figures. Mean variation of figures was 1.3 (SD =1.5) among those who experienced variation of body size.

### Changes in Binge Eating Episodes and HRQoL

A Wilcoxon signed rank test showed a significant change in binge eating episodes and selected domains of HRQoL (**Table 3**), with a medium effect. A significant change in objective binge eating episodes was observed from baseline with a median of 15,0, 95% CI (13.9-21.3) episodes to a median of 14.0 episodes, 95% CI (10.8-17.6) at end of treatment (p-value 0.02), Z = -2,33, p 0,02, r = .41. Baseline values in HRQoL in the domain “feelings” (Mdn = 4,0) improved after the intervention (Mdn = 3,0), Z = 2,68, p < 0,05, r = .44. Further, improvements of “social activities” were reported Z = -2,63, p < 0,05, r = .42, post treatment. A summary of the outcomes is presented in **Table 3**.

**Table 3:** Change in binge eating and HRQoL.

Variables	N	Pre	Post	Z	Effect size	P-value
		Mdn (IQR)	Mdn (IQR)			
**Objective binge episodes^a)^**	34	15 (11)	14 (15)	-2,33	0,41	0,02
**HRQoL^b)c)^**						
** physical fitness**	37	3 (1)	3 (1)	-1,21	0,20	0,23
** feelings**	37	4 (1)	4 (2)	-2,68	0,44	<0,05
** daily activities**	38	3 (2)	3 (2)	-0,74	0,12	0,46
** social activities**	38	3 (2)	3 (3)	-2,63	0,43	<0,05
** changes in health**	38	3 (0)	3 (1)	-1,38	0,22	0,17
** changes in overall health**	37	4 (1)	4 (1)	-0,96	0,16	0,34

Pre-post data given as median (Mdn) with interquartile range (IQR. Changes calculated from Wilcoxon signed rank test. ^a)^from the The Eating Disorder Examination-Questionnaire-6.0. ^b)^ Health related quality of life. ^c)^Ordinal levels of HRQoL with a five-point ordinal scale ranging from 1 (“no limitation at all”) to 5 (“severely limited”).

## Discussion

In this study, we described the rationale, design, and the feasibility of the People Need People (PnP) intervention for patients with obesity and comorbid BED in a Norwegian tertiary outpatient setting in Norway. This pilot study was an open label pilot from a naturalistic setting to evaluate feasability by tracking attendance and patient attrition of a study population that is representative of the population at large. Symptom severity of ED behaviors and psychological difficulties hypothesized to be associated with both BED and development of obesity was reported in addition to change in binge eating episodes and health related quality of life (HRQoL) following the intervention.

Consistent with other studies, our study showed significant overvaluation of weight and shape and eating-disorder psychopathology (132–134). In total, patients reported considerable ED psychopathology and impairment comparable to patients with ED treated in inpatient and outpatient specialized clinics in Norway (135). Patients with BED are often not given access to treatment as the diagnosis is not recognized as a distinct eating disorder diagnosis in the ICD-10 (136). Further, no or few treatment providers for obesity in Norway offer treatment for patients with obesity with comorbid BED. Given the high levels of symptom severity in our study, it is likely that a large number of patients with obesity also suffer from BED as reported from others (15–17), and thus likely to regain weight post-surgery (29, 30). This is of concern, as there are no treatment options available for patients with BED post bariatric surgery in Norway. As comorbid mental illness is associated with weight regain, postoperative complications, and adverse psychosocial outcomes in bariatric obesity populations (137), assessment and treatment of BED, including childhood trauma and underlying constructs such as shame and alexithymia, may be of relevance for selection of candidates and surgery outcomes.

Generally, drop out from ED treatment is high with dropout rates between 17–30% for binge eating disorder (BED) treatment (138). The low dropout rate in this study is optimistic compared to other interventions for obesity and comorbid BED (139–141). However, the intervention which was given in this study was short, requiring longer follow up time for a better understanding of attrition.

Alexithymia is often characterized by difficulties with emotion regulation (142). Patients with ED across diagnostic types report less emotional awareness (143) compared to individuals without ED. Difficulties with emotional regulation is consistently shown in populations with ED (144), which corresponds to our findings showing high levels of alexithymia. Research indicates that childhood trauma increases the risk for alexithymia, where both factors increase the risk for eating disorder behaviors (145). In addition to high levels of alexithymia in our study, a majority reported their childhood to be difficult or very difficult, which is in line with other studies (146). Several of the study participants described their family environment where feelings were unacceptable (e.g., children could not show negative emotions, or they would not cry or show pain/weakness) suggesting an increased risk for development of alexithymia and later binge eating behavior to regulate their emotions. Our results showing high levels of poor quality of childhood is previously shown in a systematic review by of Palmisano, Innamorati (147) indicating adverse life experiences as a risk factor for developing both obesity and BED. The fact that the large proportion of patients reported their childhood to be of poor quality is in line with the evidence linking adverse childhood experiences such as physical abuse and/or sexual abuse to obesity (148, 149) and BED (150), further supported from other studies with ED populations (84, 151, 152). Types of childhood trauma has not been associated with BMI or binge eating behavior (151), hence it may be important to consider any childhood experiences in childhood or adolescence in assessment of BED. For instance, countless episodes of bullying were reported by the participants, and suggested to have had an impact on their binge eating behavior.

The prevalence of BED in bariatric populations is uncertain and has been reported to range from 2% to 53% (153) due to large variety of methods assessing BED in bariatric populations. Research indicate that presence of post-operative binge eating is a negative predictor of weight loss outcomes (154). It may be central to assess both BED and underlying psychological constructs including the role of habit, behavioral clusters, emotion dysregulation, mood, health literacy, and executive function as interconnected drivers of BED and obesity relevant to the field of bariatric psychology (73). Patients in this study with a previous history of bariatric surgery displayed high levels of ED symptoms including grazing and binge eating behaviors similar to previous studies showing substantial patterns of disordered eating in bariatric surgery patients (153) often associated with adverse surgical outcomes, weight regain and low quality of life (155, 156). The overall reliability of the scale measuring grazing was 0,69, and highest in the sub-scale measuring compulsive eating (α =0,77), and lowest in the subscale measuring repetitive eating (α =0,52). Thus, it may be that the items in the sub scale “compulsive eating” is measuring the feeling of loss of control in relation to grazing.

The diagnostic criteria for BED does not require a patient to meet a criterion related to body image (157) such as criteria for anorexia nervosa and bulimia nervosa. Few studies have investigated body image disorder or the misperception of one’s own body in clinical patient samples with BED. Contrary to previous studies that demonstrated that individuals with obesity and BED perceive their body size as larger than their actual size (158), the majority of individuals in the current study perceived their body size accurately. They did however exhibit high levels of body disturbance and dissatisfaction, consistent with earlier findings (159). The extreme levels of poor body image among the patients in our study is consistent with finding from Dalle Grave, Calugi (140), and may suggest that symptoms involving overvaluation of shape and weight, or related constructs of body image such as perception of one’s body of body image, may be central for assessment and treatment of patients with BED (157) helping clinicians within both fields refine their procedures of assessment and treatment towards a more personalized treatment of obesity and BED. Our findings are further supported by studies using network analyses showing overvaluation of weight and shape to be central in patients seeking treatment for BED (40, 77). Further, our results may suggest that a large discrepancy between perceived and ideal body may be a significant barrier for recovery of BED and obesity. Patients with a history of bariatric surgery have shown to display poor body image at longer term follow-up as shown in a recent review by Jumbe, Hamlet (160). Hence, a deeper understanding of why patients develop poor body image pre and post-surgery may enable a better comprehension of psychological aspects of bariatric surgery and how body image is related to BED and obesity.

Given the significant presence of overvaluation of weight and shape in this patient population, it is possible that that experiences of shame may contribute to such challenges. The high levels of internalized shame found in this study may suggest that binge eating behavior is a way to cope with shame experiences, which is also found in other studies as a way to regulate emotional states (161). It may be that shame experiences for this patient population include extreme presence of overvaluation of weight and shape. Mean scores of internalized shame in our study was higher than reported in Norwegian non-clinical populations and clinical outpatient samples presenting with anxiety and depression (112). During the 10 week intervention, patients consistently described experiences of being shamed for their weight, which may have contributed to high levels of internalized shame. This is consistent with numerous other studies where weight stigma and being bullied due to weight or appearance have been reported (38, 162). A number of patients in our study reported a history of bullying because of their weight or appearance severely affecting their quality of childhood, with interweaving experiences of weight stigma, shame, poor body image and binge eating behavior through a life span.

Generally, the changes in binge eating episodes and HRQoL were positive and underline the potential of treatment targeting binge eating and grazing as a response to common stressors associated with obesity such as weight stigma, alexithymia, childhood trauma and internalized shame. The results indicate that the PnP program is feasible for patients with patients with obesity and comorbid BED, including patients with past bariatric surgery who do not meet the diagnostic criteria for BED. The intervention may influence a pathway for patients with obesity and comorbid BED before and after bariatric surgery in order to optimize treatment outcomes.

Further need of development of PnP to optimize it for individuals with high ED psychopathology became obvious during the pilot, and modifications to PnP has been implemented after the pilot study period. Several patients reported that 10 sessions were insufficient for being able to reduce BED symptoms and to incorporate knowledge and skills into their everyday life during the 10-week treatment period. Consequently, add-on therapy group interventions using principles from the 10 sessions were developed where the aim is to utilize information learned in the modules and practicing other ways to respond to stressors without binge eating. For example, body-oriented therapy sessions adapted to higher weight bodies were designed and piloted for patients who are in need of interventions targeting overvaluation of weight and shape. An additional pilot study has been approved to assess reduction of BED symptoms and increased HRQoL with lengthened treatment. This is in line with studies showing that lengthened treatment of binge eating disorders predict better outcomes (163).

### Strengths and Limits

The relatively small number of patients in the study is a limitation and our study may thus not be representative of patients with obesity and comorbid BED. Furthermore, due to the study design, we were furthermore unable to study effects of the PnP treatment. Another limitation of the current study was that recruitment occurred from a single study site; hence the patient population may be more homogeneous than other sites in Norway and what may be enrolled in a larger clinical study. Future studies will need larger sample sizes and the use of covariate analyzes to further investigate this intervention.

Strengths of the study included use of an objective measurement of BMI and validated diagnostic assessment of BED, as well as detailed description of those who did not receive the intervention or opted out. Given that the study was done in a naturalistic setting, results may be more applicable and generalizable to clinical practice compared to RCTs, hence increasing external validity of results (164). Strengths also include severity at baseline, change of binge eating episodes and HRQoL following the intervention, the use of standardized measures and regular clinical supervision.

## Conclusion

In summary, the current study provides initial support for the feasability of a brief 10‐session psychoeducational group intervention for patients with obesity and comorbid BED. It appears that emphasis on creating a safe context to explore underlying mechanisms and responses to internal and external stressors such as weight stigma, shame and poor body image may have contributed to the feasability of the study and the high attendance.

If the benefits of PnP can be reproduced, the consequence can be that patients can be treated effectively and cost-effectively, opening access to treatment for this group of patients. The content of PnP may also be of relevance and clinical importance for other groups of patients with obesity and disordered eating patterns not meeting the criteria for BED. Longer and enhanced versions of PnP may increase health outcomes and HRQoL.

A phase 2 study is being planned to establish efficacy for patient outcomes using the PnP intervention using a multiple-arm study design (165). Acceptance and tolerance will be reported in other separate study with qualitative interviews.

## Data Availability Statement

The datasets presented in this article are not readily available because availability of data can be requested to the Data Access Committee (DAC) at the Health Trust of Nord-Trøndelag, Norway. Requests to access the datasets should be directed to postmottak@hnt.no.

## Ethics Statement

The studies involving human participants were reviewed and approved by REC Central Møre og Romsdal, Sør-Trøndelag and Nord-Trøndelag. The patients/participants provided their written informed consent to participate in this study.

## Author Contributions

TE-N and KB conceived the study. TEN- oversaw overall direction and planning and took the lead in writing the manuscript. All authors provided critical feedback and helped shape the analysis and manuscript.

## Conflict of Interest

The authors declare that the research was conducted in the absence of any commercial or financial relationships that could be construed as a potential conflict of interest.

## Publisher’s Note

All claims expressed in this article are solely those of the authors and do not necessarily represent those of their affiliated organizations, or those of the publisher, the editors and the reviewers. Any product that may be evaluated in this article, or claim that may be made by its manufacturer, is not guaranteed or endorsed by the publisher.

## Footnotes

1
*We use the term «obesity» to be consistent with the medical literature. Communication about body size with patients specifically avoided the term “obese”, as studies consistently report finding the term as stigmatizing.*
